# Plants That Eat Animals

**Published:** 1882-02

**Authors:** 


### PLANTS THAT EAT ANIMALS.

WE HAVE all heard of animals that eat plants, but only recently have we learned of plants that live on animals. By recently I mean within the last twenty years. That these queer plants do really devour very small insects is now an established fact of natural history.

In 1860 Mr. Darwin, a man who studies these wonderful things and then very kindly publishes books about what he has learned, so that other people may also study them, began to notice some curious things about these singular plants, and then turned his attention to a study of them. He spent a great many hours watching the plants and their behavior. Day after day he would continue till finally he had the pleasure of knowing so much about the subject that he put it in a book.

Many other naturalists have been studying these curious plants both before and since the publication of Mr. Darwin's book. In New Jersey, North Carolina, and other States, as well as in England, these plants are to be found. There are several varieties of them, but I have room in this article to give you an account of them in only a general manner, and very briefly at that.

The leaves of these plants form a kind of mouth, and this mouth the plants can open when they choose. The leaves are the lips, and inside the mouth is a sort of throat. This throat extends downward toward the body of the plant, and has inside it a great many little bits of very short beard, which are close together and very fuzzy. In some throats are at least two hundred of these little hairy bits. At the end of each of these is a sticky substance somewhat like common mucilage.

Now, when the plant opens his mouth, you see the trap is already set. All he has to do is to keep his mouth open. By an accident a fly alights on the plant—or a bug, or some other small insect—and the moment it does so sticks fast to the gummy substance the plant is all the time making, and is caught. Fly off he cannot, and the more he tries to move the more he fastens himself to the sticky substance. The plant is now sure of a meal, for the moment a fly, a bug, or other substance, touches the throat, the mouth of the plant begins to shut, the leaves, or lips, immediately close over the victim and strangle him to death.

Then the plant eats the insect very much as you eat a bit of meat. The way you and I eat is this: We swallow the food, and the stomach digests it for us by means of what we call gastric juice. This gastric juice is a sour and bitter liquid, which cuts the food we have swallowed up, and makes pulp of it. Then the glands of our stomachs absorb, or drink up, this fluid, and it goes into the blood and gives us health and strength.

In the same way Mr. Plant eats the fly or bug. A sort of sour and bitter juice starts from the leaves on the inside and cuts the bug into pieces, and then into a sort of jelly; and after this the glands, of which the plant is very full, suck this jelly or liquid up and carry it all over the plant, and give life and strength and vigor to all its parts.

As soon as one insect is eaten in this way, the leaves open again and the trap is set for another. How many an enterprising plant of average industry will catch and devour in a day I do not exactly know. I suppose it depends on the number of them in the air.

The plants are not over particular about their diet, and will take beef, an egg, gelatine and many other things of that nature. At the same time, they have some choice about their food, and will not eat grass, starch, oil, or anything that is too fatty. A gnat tickles their palate, as turtle does the tongue of an epicure; and as for a dainty, tender butterfly, why it is said that you could not please the plant better than by feeding one to it.

So delicately is the plant made, and so sensitive are the hundreds of beards that line its throat, that a piece of human hair, one eight-thousandth part of an inch long, dropped on the plant will cause it to close immediately.

---

Effect of Good Company.—Nothing makes a man so in love with purity as purity. Many a man has been lifted out of debasing sins against which he has vainly struggled by coming to know and love a pure, sweet woman. It is the sight of embodied goodness that makes us want to be good. Many a mother by the usefulness of her life fills her children with a desire to be like her, that makes them in their turn unselfish. There are obscure men and women who hardly in their lives utter a word of conscientious teaching, who, by their example, do more to make people around them gentle, truthful and Christlike than any preacher can do. It is not those who talk about goodness, but those who are good that are the light of the world.

A gentleman recently asked a mite of a girl how old she was. "Old!" exclaimed the child, lifting her eyebrows in surprise. "Old! I'm not old at all. I'm quite new. Grandma is old."

